# Exercise Intolerance in Post-Acute Sequelae of COVID-19 and the Value of Cardiopulmonary Exercise Testing- a Mini-Review

**DOI:** 10.3389/fmed.2022.924819

**Published:** 2022-07-22

**Authors:** Álvaro Aparisi, Raquel Ladrón, Cristina Ybarra-Falcón, Javier Tobar, J. Alberto San Román

**Affiliations:** ^1^Unidad de Cardiología Intervencionista, Servicio de Cardiología, Hospital del Mar, Barcelona, Spain; ^2^Biomedical Research in Heart Diseases (GREC), Instituto Hospital del Mar de Investigaciones Médicas (IMIM), Barcelona, Spain; ^3^Servicio de Cardiología, Virgen del Rocío, Sevilla, Spain; ^4^Servicio de Cardiología, Hospital Clínico Universitario de Valladolid, Valladolid, Spain; ^5^Centro de investigación Biomédica en Red de Enfermedades Cardiovasculares (CIBERCV), Madrid, Spain

**Keywords:** post-acute sequelae COVID-19, cardiopulmonary exercise testing, autonomic dysfunction, exercise intolerance, hyperventilation

## Abstract

Coronavirus disease (COVID-19) is an infectious disease caused by the severe acute respiratory syndrome coronavirus 2 (SARS-CoV-2), with systemic organ damage in the most severe forms. Long-term complications of SARS-CoV-2 appear to be restricted to severe presentations of COVID-19, but many patients with persistent symptoms have never been hospitalized. Post-acute sequelae of COVID-19 (PASC) represents a heterogeneous group of symptoms characterized by cardiovascular, general, respiratory, and neuropsychiatric sequelae. The pace of evidence acquisition with PASC has been rapid, but the mechanisms behind it are complex and not yet fully understood. In particular, exercise intolerance shares some features with other classic respiratory and cardiac disorders. However, cardiopulmonary exercise testing (CPET) provides a comprehensive assessment and can unmask the pathophysiological mechanism behind exercise intolerance in gray-zone PASC. This mini-review explores the utility of CPET and aims to provide a comprehensive assessment of PASC by summarizing the current evidence.

## Graphical Abstract

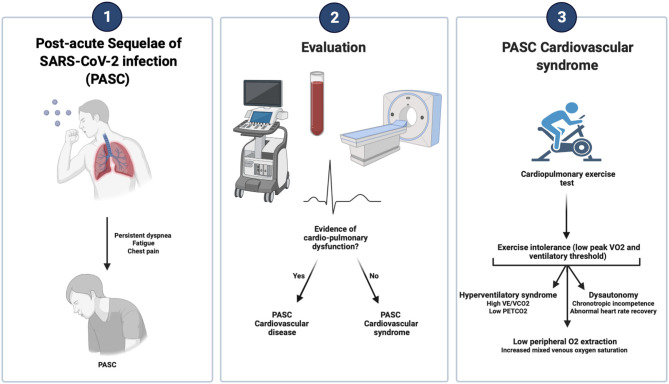

## Introduction

Long Coronavirus disease 2019 (COVID-19) or post-acute sequelae of severe acute respiratory syndrome coronavirus 2 infection (PASC) is expected to increase in prevalence and become a public health problem (1, 2). PASC is a heterogeneous clinical syndrome. The growing scientific evidence recognizes PASC as one of the conditions that cause exercise intolerance (2), but the relationships between severe acute respiratory syndrome coronavirus 2 (SARS-CoV-2) and exercise capacity remain unclear. A diminished exercise capacity has been associated with a poor quality of life and higher mortality in other conditions. Therefore, understanding the mechanism behind the limitation in exercise capacity of these patients is a fundamental step in improving patient outcomes.

A hallmark of exercise intolerance is dyspnea and fatigue upon exertion. Although it is intuitive to think that patients with PASC would be limited primarily by the cardiopulmonary system, studies indicate that most of these patients have (2) cardiac and pulmonary testing within normal values (3, 4). Exercise is dependent on the balance between oxygen supply, oxygen consumption, and clearance of toxic metabolites. These processes rely on the cardiovascular and pulmonary systems to achieve optimal exercise performance. Therefore, by studying external respiration in response to exercise, it is possible to address the functional competence of the organ systems by coupling external adjustments to cellular respiration. Cardiopulmonary exercise testing (CPET) offers the opportunity to study the cellular, cardiovascular, and ventilatory systems' responses simultaneously, providing an objective evaluation of exercise capacity (5, 6).

This contemporary review focuses on the essential role of CPET in the evaluation of patients with PASC and the potential mechanism behind exercise intolerance. Therefore, we explore and summarize the currently available evidence to increase awareness of this entity and improve the quality of care.

### Post-Acute Sequelae of SARS-CoV-2 Infection

PASC represents the long-term sequelae of COVID-19 and it is classified according to the time frame of symptom persistence into a subacute (4–12 weeks) or chronic phase (>12 weeks) (1). PASC occurs in a heterogeneous group of patients with different clinical presentations (2), but it is characterized by a systemic involvement with the ability to impair patients' quality of life (4). Recent studies have looked at risk factors contributing to PASC observing an association with symptom burden during an active infection, female gender, and COVID-19 severity (7, 8).

The American College of Cardiology classifies PASC into two groups whether there is objective evidence of cardiovascular disease. Accordingly, PASC-cardiovascular disease is characterized by myocardial, pericardial, vascular, and/or arrhythmic conditions that appear beyond 4 weeks from the initial SARS-CoV-2 infection. Whereas, the term PASC-cardiovascular syndrome (PASC-CVS) is defined by the absence of cardiovascular disease, but on the contrary, also by the persistence of cardio-pulmonary symptoms. The two most commonly reported symptoms are fatigue and dyspnea, regardless of PASC time (9). Both are common in non-COVID patients with other cardiopulmonary conditions (10, 11) and in the convalescence phase of any critical illness (12), where exercise intolerance is also a characteristic feature (10, 11). Therefore, we should expect a high prevalence of exercise intolerance among COVID-19 survivors. Data regarding the pathophysiologic mechanism behind PASC-CVS are scarce, but it is not yet fully understood how this translates into reduced exercise capacity.

### Evidence of Exercise Capacity in PASC-CVS

Despite there are no dedicated guidelines on the evaluation of PASC-CVS, patients should undergo a CPET evaluation to identify limiting factors for decreased maximal exertion that is usually found during the convalescence phase of any critical illness (12). Otherwise, the lack of evidence of residual cardiopulmonary damage may cause a delay in the diagnosis. Exercise capacity is evaluated through the assessment of oxygen consumption (Vo_2_) at peak exercise if a sustained maximal effort has been reached. Maximal performance is also age-, sex- and weight-adjusted to be reported as a percentage predictive of peak Vo_2_. Moreover, ventilatory threshold (V_T_) is effort-independent providing a more accurate assessment of aerobic efficiency and reflecting the Vo_2_ at submaximal exercise levels when supply does not match requirements triggering an anaerobic environment (5, 6).

Follow-up studies on SARS-CoV survivors observed a diminished exercise capacity (13, 14), but similar findings were observed during hospital discharge because of COVID-19 (15). To date, there is only scarce observational data on exercise capacity with PASC-CVS during follow-up. A small single-center retrospective study found during short-term follow-up a diminished exercise capacity (16). Similar findings were reported by a multi-center retrospective study of 200 patients, in which those with PASC (56%) had a lower peak Vo_2_ (25.8 ± 8.1 vs. 28.8 ± 9.6 mL/min/kg; *p* = 0.017) and a smaller chance of achieving the V_T_ (OR: 0.38; 95% CI 0.20–0.72) (17). On the contrary, mid-to-long-term retrospective studies identify border-line exercise capacity in PASC patients (18) that was not different from controls with unexplained dyspnea (19).

These data were reproduced by prospective studies during short-term follow-up (20–24) and when stratified by COVID-19 severity no differences were found between groups (25); however, other studies reported that previous critical care unit admission, need for mechanical ventilation and a longer hospital stay were independently associated with peak Vo_2_ (26). The incorporation of unexplained dyspnea in PASC-CVS into the design and analysis of recent studies have yielded similar findings during follow-up. A single-center prospective study of 70 consecutive patients observed that PASC-CVS patients with persistent dyspnea (59%) experienced a smaller exercise capacity (78 vs. 99% of predicted peak Vo_2_; *p* < 0.001) than asymptomatic COVID-19 survivors (27). Accordingly, a multicenter prospective study that evaluated 156 patients also reported among PASC patients with persistent dyspnea (47%) a diminished exercise capacity (76 ± 16 vs. 89 ± 18% of predicted peak Vo_2_; *p* = 0.009) (28).

An unexplored scenario is the possibility of an immediate improvement in the functional capacity of PASC patients. A prospective study that monitored the persistence of exercise intolerance in PASC with serial CPET evaluation reported an improvement between 3-and-6 months of peak Vo_2_ (18 vs. 20.5 mL/kg/min; *p* = 0.001) and V_T_ (9.7 vs. 10.4 mL/min/kg; *p* = 0.018). However, these improvements were not observed in all patients and were less evident when compared to healthy controls (29). Findings from other prospective studies confirm that exercise intolerance is also observed during mid-to-long term follow-up in PASC (30–35).

In general, low peak Vo_2_ is common among patients with PASC-CVS during follow-up (Table 1), but application and interpretation of CPET results are challenging. Peak Vo_2_ is defined by the Fick equation as the product of cardiac output and arteriovenous oxygen difference [C (a–v) O2]. This is important because cardiac output is the product of stroke volume times heart rate (HR) and arteriovenous oxygen difference reflects the peripheral oxygen tissue extraction (5, 6). Consequently, abnormalities in any of these variables can contribute to exercise intolerance in PASC-CVS.

**Table 1:** Most Relevant Studies evaluating exercise capacity in Post-acute sequelae of COVID-19 with cardiopulmonary exercise test.

**References**	**Study design**	**Sample size**	**Follow-up**	**Main findings**
Baratto et al. (15)	Single center	36	Hospital discharge	COVID-19 patients had at the time of discharge a smaller exercise capacity (59 vs. 90% of predictive peak Vo_2_; *p* < 0.001) and peripheral oxygen extraction (0.66 vs. 0.81; *p* = 0.006) compared to controls^a^. COVID-19 patients had ventilatory inefficiency (VE/VCO_2_ slope 32 vs. 28 mmHg; *p* = 0.007) likely explained by hyperventilation. Ca_O2_ (tau = 0.58, *p* = 0.012) and hemoglobin (*R* = 0.46, *p* = 0.002) were positively correlated with peak Vo2. No differences were found in cardiac echocardiography at rest between groups.
Mohr et al. (16)	Single center retrospective	10	3 months	PASC patients with persistent dyspnea showed a mean 72.7% of predictive peak Vo_2_, 78.1 ± 7.3% of predictive heart rate, 96 ± 15.5% of predictive Vo_2_/HR and a mean lactate post-exercise of 5.6 ± 1.8 mmol/L.
Barbagelata et al. (17)	Multicenter retrospective	200	3 months	PASC patients^b^ showed a lower peak Vo_2_ (25.8 ± 8.1 vs. 28.8 ± 9.6 mL/min/kg; *p* = 0.017) but with a similar % of predicted peak Vo_2_ (89.7 ± 19.9 vs. 92.9 ± 18.7 %; *p* = 0.257) compare to asymptomatic post-COVID patients. Ventilatory efficiency was similar between groups (VE/VCO_2_ slope 33.1 ± 5.9 vs. 32.5 ± 5.5; *p* = 0.521). Most common reported symptom during CPET was dyspnea^c^ (93%), particularly in PASC patients (97 vs. 75%; *p* = 0.008). PASC was associated with smaller V_T_ (OR: 0.38; 95% CI 0.2–0.72) and a greater chance of symptoms during CPET (OR: 7.0; 95% CI: 3.5–16.2).
Debeaumont et al. (18)	Single center retrospective	23	6 months	Persistent dyspnea^c^ was significantly associated with peak Vo_2_ (rho = −0.49). PASC was associated with a diminished % of peak Vo_2_ (84 ± 19%), particularly in ICU survivors (77 ± 15 vs. 87 ± 20%). Ventilatory efficiency was low (VE/VCO_2_ slope 32 ± 5) in the global cohort, but higher in ICU survivors (VE/VCO_2_ slope 34 ± 5). Hemoglobin and pulmonary function test were within normal reference values.
Alba et al. (19)	Single center retrospective	36	8 months	PASC patients with persistent dyspnea^b^ had comparable peak Vo_2_ (20 vs. 19.5 mL/min/kg; *p* = 0.8), % of predicted peak Vo_2_ (85.5 vs. 85%; *p* = 0.9), anerobic threshold and ventilatory efficiency (VE/VCO_2_ slope 29.8 vs. 28.4; *p* = 0.15) compare to controls^d^. PASC patients with abnormal CPET where mostly characterized by low O2 pulse with a normal cardiac function suggestive of a peripheral limitation. One patient underwent iCPET that showed a high mixed O2 venous content. Hemoglobin and pulmonary function test were within normal values.
Szekely et al. (20)	Single center prospective	106	3 months	PASC patients (67%) had a lower V_T_ (12.3 ± 3.6 vs. 15.4 ± 5.7 mL/min/kg; *p* = 0.02) and Vo_2_ (1.6 ± 0.5 vs. 2.24 ± 0.9 L/min; *p* = 0.03) compared to controls^e^ (33%). PASC patients had a smaller CO (9.8 ± 2.7 vs. 14 ± 4.2 L/min; *p* < 0.0001) and greater A-Vo_2_ difference (0.18 ± 0.05 vs. 0.13 ± 0.04; *p* = 0.004) compared to controls^e^ suggesting a cardiac limitation. PASC patients with persistent dyspnea showed ventilatory inefficiency (VE/VCO_2_ slope 30.5 ± 4 mmHg) and chronotropic incompetence.
Ribeiro Baptista et al. (21)	Single center prospective	105	3 months	35% of patients with previous severe COVID-19 had a diminished exercise capacity defined by <80% of predicted peak Vo_2_. Impaired exercise capacity was associated with decrease lung volumes and D_LCO_, but with a preserved breathing reserve at peak Vo_2_. Cardiac dysfunction at rest was not observed at rest, but those with diminished exercise capacity had a smaller % predicted Vo_2_ /HR (66 ± 9.6 vs. 96.6 ± 14.7; *p* < 0.0001) suggestive of peripheral limitation.
Clavario et al. (22)	Single center prospective	200	3 months	59% of patients complained about dyspnea^d^ and the global cohort showed a median of 85 (74–98) % of predicted peak Vo_2_. Main causes of exercise limitation were non-cardiopulmonary (50.8%) among patients with <85% of predicted peak Vo_2_ (50.5%). Pulmonary lung function in the entire cohort, but those <85% of predicted peak Vo_2_ showed a smaller % of predicted D_LCO_ (70 vs. 85%; *p* < 0.001). Predicted FEV1 (95% CI: 0.73–9.85, *p* = 0.023), D_LCO_ (95% CI: 2.49–10.13, *p* = 0.001), and dominant leg extension maximal strength (95% CI: 3.83–24.35, *p* = 0.008) were independently associated with peak Vo_2_.
Rinaldo et al. (23, 25)	Single center prospective	75	3 months	Most common reported symptom was dyspnea^b^ (52%). Average peak Vo_2_ was 20 mL/min/kg that corresponded to 83 ± 15% of the predicted peak Vo2, no differences were observed irrespective of previous COVID-19 severity (*p* = 0.895). Average VE/VCO_2_ slope was 28.4 ± 3.1 and the median alveolar–arterial gradient for oxygen was 26 (18–31) mmHg. Pulmonary lung function test was within normal mean values, but D_LCO_ was diminished irrespective of the exercise capacity (74 ± 14 vs. 69 ± 13% *p* = 0.175). Mean hemoglobin level was 15.0 ± 1.5 g/dL.
Jahn et al. (24)	Single center prospetive	35	3 months	Pulmonary function and D_L_co were normal with values ≥80% of predicted in 66% of patients despite previous severe COVID-19^f^. 46% of patients had ≥82% of predicted peak Vo_2_ and 54% had <81% of predicted peak Vo_2_. Patients with a <82% of predicted peak Vo_2_ had a smaller % of predicted D_LCO_ (80 ± 13 vs. 96 ± 18%; *p* = 0.06). Exercise limitation due to neuromuscular impairment was considered unlikely given the normal maximal inspiratory (99.4% of predicted) and expiratory (79.9% of predicted) pressures.
Motiejunaite et al. (26)	Single center prospective	114	3 months	Most common reported symptom was dyspnea (40%) and fatigue (32%). Entire cohort had a diminished exercise capacity (71% of predicted peak Vo_2_, but those with a D_LCO_ ≤ 75% (42%) had a smaller % of predicted peak Vo_2_ (16.2 vs. 19 mL/min/kg; *p* < 0.001) and V_T_ (39 vs. 45%; *p* = 0.014). Median VE/VCO_2_ slope was 33, and irrespective of D_LCO_ ≤ 75% (VE/VCO_2_ slope 34 vs. 32; *p* = 0.105), suggesting a ventilatory inefficiency. Inappropriate hyperventilation was observed in 24% of all patients. No differences were observed in resting echocardiography. Age, ICU admission, mechanical ventilation and length of hospital stay were independently associated with % predicted peak Vo_2_.
Aparisi et al. (27)	Single center prospective	70	3 months	Persistent dyspnea^g^ was associated with a diminished QoL (*p* < 0.001), exercise capacity (77.8 vs. 99% of predictive peak Vo_2_; *p* < 0.001) and ventilatory inefficiency (VE/VCO_2_ slope 32 vs. 29.4 mmHg; *p* = 0.022). Need of hospital admission was not associated with a greater rate of persistent dyspnea (*p* > 0.05) during follow-up. No differences were observed between groups in resting echocardiography, laboratory makers and pulmonary lung function. No signs of pulmonary embolism or fibrosis among those who underwent CT-scans.
Skjørten et al. (28)	Multicenter prospective	156	3 months	Patients with persistent dyspnea^a^ had a lower % of predictive peak Vo_2_ compared to asymptomatic patients (76 ± 16 vs. 89 ± 18 %; *p* = 0.009), but without abnormalities in lung function, breathing reserve, peripheral O2 and D_LCO._ Patients with persistent dyspnea were characterized by ventilatory inefficiency mostly due to circulatory limitation (38%) and dysfunctional breathing pattern (46%). Those with previous ICU admission showed during follow-up a smaller exercise capacity (82 ± 15% vs. 90 ± 17% of predictive peak Vo_2_; *p* = 0.004) compared to non-ICU patients.
Cassar et al. (29)	Single center prospective	88	6 months (serial assessment)	PASC patients (previous history of moderate-severe COVID-19) had a significant smaller exercise capacity during 3 (peak Vo_2_ of 18 vs. 28 mL/kg/min; V_T_ of 9.7 vs. 11.9 mL/min/kg) and 6 (peak Vo_2_ of 20.5 vs. 28 mL/min/kg; V_T_ of 10.4 vs. 11.9 mL/min/kg) months follow-up compare to controls^h^. Ventilatory response was abnormal (VE/VCO_2_ slope >30) regardless the time-frame compare to controls. Heart rate recovery was impaired at 3 months (16.6 vs. 21.9 bpm; *p* = 0.018), but improve at 6 months (22.2 vs. 21.9 bpm; *p* = 0.67) compare to controls. No differences during serial cardiac imaging were observed. Hemoglobin was within normal values. There was no correlation between the extent of lung abnormalities on MRI, lung function parameters and dyspnea.
Dorelli et al. (30)	Single center prospective	28	6 months	Patients with ventilatory inefficiency (28.6%) had a smaller HR recovery (17.5 ± 7.6 vs. 24.4 ± 5.8; *p* = 0.015), but with similar peak Vo_2_ (32.9 ± 13.1 vs. 27.6 ± 5.2; *p* = 0.137) to those without ventilatory inefficiency. No differences were observed in pulmonary lung function between groups. Ventilatory inefficiency was inversely correlated with HR recovery (*r* = −0.537; *p* = 0.003).
Vannini et al. (31)	Single center prospective	41	6 months	Most common reported symptoms were dyspnea (56.1%) and fatigue (51.2%), with a similar prevalence irrespective of exercise capacity. Mean % of predictive peak V_O2_ was 73.6 ± 15.6 %, without differences according to previous disease severity (*p* > 0.05) despite severe pneumonia and ARDS presented lower D_LCO_ in comparison to mild pneumonia (6.85 vs. 7.72 vs. 9.35 mmol/min*kPa; *p* = 0,04 and *p* = 0.033). Basal and stress test echocardiographic findings were within normal values. 36.5% of the patients exhibit an abnormal ventilatory response (VE/VCO_2_ slope >30) to exercise without significant desaturation or pathological Vd/V_T_ increase.
Ladlow et al. (32)	Single center	205	6 months	25% of the patients met the criteria for dysautonomia^i^, this group had lower Vo_2_ at V_*T*_ (12.6 ± 2.1 vs. 14.1 ± 3.2 mL/kg/min; *p* = 0.001) and peak exercise (30.6 ± 5.5 vs. 35.8 ± 7.6 mL/kg/min; *p* = 0.001). PASC patients with dysautonomia had a higher HR at rest (95 ± 12 vs. 81 ± 12 bpm; *p* < 0.001) and in the first V_T_ (114 ± 15 vs. 107 ± 17 bpm; *p* = 0.017), but smaller HR at peak exercise (170 ± 13 vs. 177 ± 15 bpm; *p* = 0.003) and attenuated HR recovery (17 ± 4 vs. 31 ± 17 bpm; *p* < 0.001). Patients with dysautonomia showed a lower ventilatory efficiency (VE/VCO_2_ slope 29.9 ± 4.9 vs. 27.7 ± 4.7 mmHg; *p* = 0.005) and a higher breathing frequency.
Vonbank et al. (33)	Single center prospective	100	6 months	Lung function was within normal values, but D_LCO_ was lower in PASC with previous severe disease (74.8 ± 18.2 vs. 85 ± 14.8; *p* = 0.01). Compared to controls, PASC with previous mild and severe disease had a significant smaller % of predictive peak Vo_2_ and V_T_. Patients with previous severe COVID-19 showed a smaller % of predictive D_LCO_ (74.8 vs. 85%; *p* = 0.01), but other lung function parameters were comparable and within normal values. Ventilatory inefficiency (higher VE/VCO_2_) was evident among PASC compared to healthy controls^k^ at V_T_ and peak exercise. Younger age, male sex, lower BMI, higher D_LCO_ and lower breathing reserve were associated with a higher peak Vo_2._
Mancini et al. (34)	Single center prospective	41	9 months	PASC patients had an average 77 ± 21% of predicted peak Vo_2_ and 10.6 ± 2.8% of predicted Vo_2_ at V_T_. Those with peak Vo_2_ <80% of predicted had a circulatory limitation to exercise. 88% of PASC patients had dysfunctional breathing, ventilatory inefficiency (increased VE/VCO_2_ slope) and/or hypocapnia (PetCO_2_ < 35 mmHg). 46% of the patients met the criteria for myalgic encephalomyelitis/chronic fatigue syndrome.
Singh et al. (35)	Single center prospective	20	11 months	COVID-19 survivors had a smaller exercise capacity (70 ± 11 vs. 131 ± 45% of predictive peak Vo_2_; *p* = 0.001) and a greater degree of ventilatory inefficiency (VE/VCO_2_ slope 35 ± 5 vs. 27 ± 5 mmHg; *p* = 0.01) compared to controls^f^. COVID-19 survivors showed a greater peak exercise mixed venous oxygen saturation (50 ± 10% vs. 22 ± 5%; *p* < 0.0001) and peak Vo_2_ content (33 ± 6 vs. 27 ± 5 mmHg; *p* = 0.01) suggesting a peripheral limitation to aerobic exercise. No differences were observed in terms of right atrial pressure, left-side filling pressure and total pulmonary. resistance at peak exercise between groups

*BMI, body mass index; CT, computed tomography; FEV1, forced expiratory volume in 1 second; MRI, magnetic resonance imaging; Ca_O2_, content of oxygen in arterial blood; CO, cardiac output; CPET, cardio-pulmonary exercise test; D_LCO_, diffusion carbon monoxide capacity; ICU, intensive care unit; QoL, quality of life; V/Q, ventilation/perfusion; HR, heart rate; Vo_2_, oxygen consumption; Vo_2_ /HR, oxygen pulse; V_T_, Ventilatory threshold; VE/VCO_2_ slope, slope of minute ventilation to CO_2_ production*.

a *Matched age, sex and body mass index healthy controls in 1:1 ratio with COVID-19 patients*.

b *Defined as dyspnea or fatigue persisting for at least 45 days after symptom onset*.

c *Defined as mMRC >1*.

d *Matched controls also complained about unexplained dyspnea*.

e *Historical matched age, sex, weight, height, hypertension and diabetes controls*.

f *Severe COVID-19 was defined if ≥2 of the following criteria were met: respiratory rate >30 bpm, peripheral oxygen saturation <93% while breathing ambient air, C-reactive protein levels >75 mg/L, ground glass opacities or diffuse infiltrates on CT scan, or rapid progression of CT findings >50% within 24–48 h*.

g *Defined as NYHA > II*.

h *Negative SARS-CoV-2 controls matched for age, sex, body mass index and risk factors (smoking, diabetes, and hypertension) without previous hospitalization*.

i *Patients with dysautonomia met the following criteria: (1) resting HR of >75 bpm; (2) increase in HR during exercise of <89 bpm; and (3) HR recovery of < 25 bpm in the first 60 s after cessation of exercise*.

j *Symptomatic normal individuals with a normal peak Vo_2_ and peak CO of ≥80% predicted in invasive CPET*.

k *Healthy controls matched for age, sex, body mass index*.

### Contributors of Exercise Intolerance in PASC-CVS

Identification of patterns during CPET may identify the organ systems involved in the exercise intolerance referred by PASC patients as we cannot rely exclusively on a decreased peak Vo_2_ and V_T_ (see Graphical Abstract). Therefore, CPET can be combined with the invasive and non-invasive tests to further phenotype more accurately PASC-CVS. However, given the systemic nature of COVID-19, we may expect a cardiac, ventilatory, peripheral, and/or pulmonary gas exchange limitation at exercise.

### Cardiovascular Limitation

Cardiovascular limitation to exercise intolerance in PASC-CVS patients may be explained by several factors, but electrocardiographic changes and a pathological blood pressure response during exercise have not yet been reported. Moreover, low CO could explain exercise intolerance in most PASC patients; however, no left ventricular dysfunction has been reported in the studies that evaluated cardiac function at rest (15, 21, 26, 27, 29). Similarly, two prospective studies that evaluated cardiac function at rest and during CPET concluded that cardiac function was within normal values, regardless of previous COVID-19 severity (20, 31). However, Szekely et al. also observed a reduced stroke volume with a blunted peak HR and a higher peak arteriovenous difference among PASC patients (20), raising the possibility of a cardiac autonomic dysregulation as a major cause of exercise intolerance.

Modulation of the HR during exercise is a dynamic process tightly regulated by the autonomic nervous system and its imbalance may manifest during exercise as chronotropic incompetence or inadequate HR recovery. Some of the studies reported chronotropic incompetence (16, 20), while others observed an abnormal HR recovery (29, 30, 32). Interestingly, both were more commonly observed among PASC patients with evidence of ventilatory inefficiency.

### Ventilatory and Pulmonary Vascular Limitation

Lung mechanical-related mechanism because of significant reduction of pulmonary function should be, in theory, the expected primary cause of exertional dyspnea in PASC. Contrary to that, most of the studies did not observe a correlation between abnormal lung functions and persistent dyspnea regardless of COVID-19 severity. This is further supported by normal breathing reserve among PASC patients (18, 19, 23, 27, 28, 30, 33). However, some studies reported a significant decrease in D_LCO_ showed some discordant findings concerning peak V_O2_ (21, 22, 26, 29, 31, 33).

Under normal conditions, ventilation increases proportionally to CO_2_ production (36) but a common finding from the CPET of PASC patients is the ventilatory inefficiency (increased VE/VCO_2_ slope) suggesting an abnormal response (15, 17, 18, 20, 26–28, 30, 33, 35). Multiple mechanisms can explain it, but PASC may present with a characteristic pattern observed in pulmonary vascular or interstitial diseases (36). Pulmonary hypertension is typically seen with a diminished partial pressure of end-tidal CO_2_ (37), which has also been reported in PASC patients with ventilatory inefficiency (27, 34, 35). However, several findings argue against this hypothesis. First, despite COVID-19 being associated with pulmonary embolism or right ventricular dysfunction (38), none of the studies reported such findings in PASC patients (26, 27, 29, 31). Second, those studies with stress test echocardiogram or invasive CPET did not observe signs of exercise-induced pulmonary hypertension with exception of a few isolated cases (34, 35). Third, an increase in the physiological dead space/tidal volume ratio was not observed (15, 18, 30, 31, 34, 35) when a raise is expected with severe ventilation-perfusion mismatching (37). Finally, no peripheral oxygen desaturation was reported even in those with pathological D_LCO_ (21–26, 33).

More recently, hyperventilation syndrome has been suggested to occur in PASC patients (26) given the increase VE/VCO_2_ and low PETCO_2_ observe during exercise without clear evidence of cardio-pulmonary diseases (39). Therefore, dysfunctional breathing characterized by exercise-induced hyperpnea may explain the persistence of symptoms in PASC-CVS.

### Peripheral Limitation

The peripheral limitation has also been postulated as contributing to PASC. Alba et al. (19) found that a great number of PASC patients with abnormal CPET showed a low O_2_ pulse with a normal cardiac function, suggesting a peripheral limitation. In the same way, Ribeiro Baptista et al. (21) didn't observe cardiac dysfunction at rest, but those with diminished exercise capacity had a smaller predicted O_2_ pulse suggestive of peripheral limitation. As already mentioned, according to the Fick equation (peak V_O2_ is defined as the product of cardiac output and arteriovenous oxygen difference), a depressed peak V_O2_ can be the result of a blunted cardiac output response (impair oxygen delivery), and impaired peripheral oxygen extraction (diffusion defect) or both (5, 6).

In this sense, Singh et al. (35) performed invasive cardiopulmonary exercise testing on 10 patients who had recovered from COVID-19. These patients, in contrast to the control group, showed an increased peak exercise mixed venous oxygen saturation and peak venous O_2_ content. The authors concluded that the impaired oxygen extraction was attributed primarily to reduced oxygen diffusion in the peripheral microcirculation, exhibiting a peripheral limitation to aerobic exercise.

Underlying anemia can contribute to both reduced systemic oxygen delivery and extraction (5, 6). Several studies collected hemoglobin levels, with the mean being within normal ranges (18–20, 23, 27, 35), ruling out the presence of anemia as a contributing factor to reduced peak V_O2_ found in these patients. However, one study from Baratto et al. (15) found underlying anemia in patients who had recovered from COVID-19 at the time of hospital discharge. This reduction in hemoglobin levels leads to reduced arterial O_2_ content and therefore to a lower O_2_ delivery and reduced peak V_O2_.

## Discussion

PASC is a disorder that occurs irrespective of previous disease severity and is characterized by a myriad of conditions and symptoms. Data suggest that dyspnea, fatigue, and exercise intolerance are the most common referred symptoms during outpatient follow-up. PASC cardiovascular disease is associated with structural or functional cardiovascular abnormalities that may explain the persistence of symptoms, but a non-negligible number of patients have no objective evidence of organ involvement. Therefore, PASC-CVS represents a heterogeneous group of patients with persistent symptoms that generally present a normal cardiopulmonary function (9). Because of a paucity of data, not much attention has been given to CPET despite its application can accurately evaluate PASC-CVS and improve the quality of care for these patients.

PASC-CVS is associated with an objective reduction of the exercise capacity during CPET (15–35). A long list of conditions can lead to poor physical conditions referred by PASC-CVS patients. Among all the potential causes and CPET variables, the following stand out: chronotropic incompetence, abnormal heart rate recovery, ventilatory inefficiency (high VE/VCO_2_ and low PETCO_2_), and diminished peripheral oxygen extraction. Patients presenting with pulmonary vasculopathy or interstitial lung disease demonstrate similar findings during CPET (36). PASC-CVS may share a common mechanism, but data from follow-up studies do not support that hypothesis as most show no evidence of cardio-pulmonary sequelae (15–35).

Therefore, given all key factors in determining oxygen availability (5, 6), it seems that the cornerstone of PASC-CVS may involve a peripheral limitation. This is further supported by invasive CPET findings, where a diminished peripheral tissue extraction during exercise led to a decreased exercise capacity (35). Notably, hyperventilation leads to a leftward shift of the hemoglobin oxygen affinity that is translated into a decreased O_2_ unloading and impaired diffusion (5, 6). However, such impaired diffusion could also be explained by direct damage to the endothelium (40) leading to exercise intolerance as observed in chronic fatigue syndrome (41). Similarly, endothelial dysfunction has been reported in PASC with and without chronic fatigue syndrome (42). Interestingly, all the aforementioned factors may be linked to autonomic dysregulation (43, 44).

Dysfunctional breathing has been widely described among PASC patients (45), with hyperventilation being characterized by a decreased exercise capacity and signs of ventilatory inefficiency without evidence of cardio-pulmonary dysfunction. Interestingly, a high respiratory rate causes sympathetic activation and vagal withdrawal leading to exercise intolerance not only through an impaired O_2_ diffusion but also through a diminished O_2_ delivery (43). The impairment in O_2_ delivery is supported by the evidence of cardiac autonomic dysfunction among PASC patients (32, 46, 47), where ventilatory inefficiency was also a common finding (16, 20). However, PASC-CVS may also manifest as dysfunctional breathing with a chaotic ventilatory pattern with normal peak VO_2_, PETCO_2_, and VE/VCO_2_ during CPET (48).

Finally, evidence of autonomic dysfunction in PASC is further supported by recent studies suggesting that some patients present with signs and symptoms suggestive of postural orthostatic tachycardia syndrome (49). Indeed, postural orthostatic tachycardia syndrome can explain the CPET findings as it is associated with sympathetic stimulation, vasoconstriction, and hyperpnea (50).

Thus, the most appropriate hypothesis seems to be cardiac and peripheral autonomic dysregulation creating a vicious cycle that alters the exercise capacity in PASC-CVS. Nevertheless, it is difficult to draw any definitive conclusions, as the observations might be time-sensitive. In addition, none of the studies reported the baseline physical activity and physiological status of individuals before getting COVID-19, which raises the possibility of a cause-effect bias.

### Prognostic Utility of CPET in PASC-CVS

PASC-CVS is expected to become a major challenge as most recent findings suggest that it shares some features with chronic fatigue syndrome (50). Although younger age and shorter time since COVID-19 have been recently described as potential predictors of submaximal CPET in PASC (51), there are no published studies examining the long-term prognostic value unless some ideas are extrapolated from previous studies. In particular, in heart failure patients a peak Vo_2_ >14 ml/kg/min is associated with smaller 1-year mortality (52). Similarly, a high VE/VCO_2_ slope is also associated with the worst clinical outcomes among cardiac and pulmonary patients (53) with recent studies suggesting that a high VE/VCO_2_ in PASC-CVS is an independent predictor for endothelial dysfunction (54). Endothelial dysfunction has been associated with the worst outcomes in other medical conditions (44). Therefore, the presence of a diminished peak Vo_2_ or high VE/VCO_2_ slope could be associated with an increased risk of death during follow-up.

Interestingly, there is growing evidence that autonomic dysfunction might a fundamental factor in the observed symptoms in PASC-CVS (49). Theoretically, we could speculate about the potential utility of HR dynamics assessment in this group of patients during maximal effort and recovery. Previous studies have noted that chronotropic incompetence is associated with poor outcomes in heart failure patients (55). Furthermore, the detection of a heart rate recovery of ≤12 beats per minute is a strong predictor of all-cause mortality (RR: 2; 95% CI 1.5–2.7; *p* < 0.001) irrespective of previous cardiovascular risk factors and even in the absence of heart failure or myocardial perfusion defects (56). Nevertheless, risk stratification for PASC-CVS is limited. Thus, future studies with CPET both at baseline and follow-up are expected and will provide a more reliable estimation of long-term clinical outcomes in these patients with a special emphasis on previously known prognostic factors.

## Conclusions

Our current understanding of PASC is vague, but exercise limitation is a common finding despite the absence of objective cardio-pulmonary sequelae in PASC-CVS. Physiological assessment with CPET may provide valuable information about the functional status of these patients and identify the potential pathogenic mechanism. Autonomic dysfunction might be the missing link. Future studies evaluating predictors of exercise intolerance and long-term prognosis are warranted, as it could have a positive effect on disease evolution and clinical outcomes.

## Author Contributions

ÁA, JT, and JASR contributed to conception of the work. ÁA wrote the first draft of the manuscript. ÁA, RL, and CY-F wrote sections of the manuscript. All authors contributed to manuscript revision, read, and approved the submitted version.

## Conflict of Interest

The authors declare that the research was conducted in the absence of any commercial or financial relationships that could be construed as a potential conflict of interest.

## Publisher's Note

All claims expressed in this article are solely those of the authors and do not necessarily represent those of their affiliated organizations, or those of the publisher, the editors and the reviewers. Any product that may be evaluated in this article, or claim that may be made by its manufacturer, is not guaranteed or endorsed by the publisher.

## Abbreviations

COVID-19: coronavirus disease 2019
CPET: cardiopulmonary exercise test
HR: heart rate
PASC: postacute sequelae of COVID-19
PASC-CVS: postacute sequelae of COVID-19 cardiovascular syndrome
Vo_2_: oxygen consumption
V_T_: anaerobic or ventilatory threshold
VE/VCO_2_: minute ventilation/CO_2_ output.
